# Long COVID Prevalence and Risk Factors: A Systematic Review and Meta-Analysis of Prospective Cohort Studies

**DOI:** 10.3390/biomedicines13122859

**Published:** 2025-11-24

**Authors:** Ramona-Georgiana Halas, Delia Mira Berceanu Vaduva, Matilda Radulescu, Ana-Cristina Bredicean, Diana-Maria Mateescu, Ana-Olivia Toma, Ioana-Georgiana Cotet, Cristina-Elena Guse, Andrei Marginean, Madalin-Marius Margan, Voichita Elena Lazureanu

**Affiliations:** 1 Doctoral School, Department of General Medicine, “Victor Babes” University of Medicine and Pharmacy, Eftimie Murgu Square 2, 300041 Timisoara, Romania; ramona.stanculete@umft.ro (R.-G.H.); diana.mateescu@umft.ro (D.-M.M.); ioana.cotet@umft.ro (I.-G.C.);; 2 Discipline of Microbiology, Department XIV Microbiology, University of Medicine and Pharmacy from Timisoara, Eftimie Murgu Sq. No. 2, 300041 Timisoara, Romania; berceanu.delia@umft.ro; 3 Department of Microbiology, Multidisciplinary Research Center on Antimicrobial Resistance, “Victor Babes” University of Medicine and Pharmacy, Eftimie Murgu Square 2, 300041 Timisoara, Romania; radulescu.matilda@umft.ro; 4 Department of Neuroscience, Discipline of Psychiatry, Center for Cognitive Research in Neuropsychiatric Pathology (NeuroPsy-Cog), “Victor Babes” University of Medicine and Pharmacy Timisoara, Eftimie Murgu Square 2, 300041 Timisoara, Romania; 5 “Dr. Victor Popescu” Military Emergency Clinical Hospital, 300080 Timisoara, Romania; andreivmarginean@yahoo.com; 6 Center for the Morphologic Study of the Skin (MORPHODERM), Victor Babes University of Medicine and Pharmacy Timisoara, 300041 Timisoara, Romania; 7 Department of Public Health and Sanitary Management, “Victor Babes” University of Medicine and Pharmacy, Eftimie Murgu Square 2, 300041 Timisoara, Romania; margan.madalin@umft.ro; 8 Discipline of Infectious Disease, “Victor Babes” University of Medicine and Pharmacy, Eftimie Murgu Square 2, 300041 Timisoara, Romania; lazureanu.voichita@umft.ro

**Keywords:** long COVID, post-acute sequelae, prevalence, risk factors, meta-analysis

## Abstract

**Background**: Long COVID, or post-acute sequelae of SARS-CoV-2 infection (PASC), affects millions globally, with persistent symptoms impacting quality of life. This meta-analysis synthesizes prospective cohort studies to estimate the prevalence of Long COVID symptoms and identify risk factors. **Methods**: We systematically searched PubMed for prospective cohort studies (2020–2025) on Long COVID, focusing on prevalence and risk factors. Studies with ≥100 participants and follow-up ≥3 months were included. Data were extracted on symptom prevalence (e.g., fatigue, dyspnoea) and risk factors (e.g., sex, hospitalization). Random-effects models were used to pool prevalence and odds ratios (OR). Risk of bias was assessed using the Newcastle–Ottawa Scale (NOS). **Results**: Fourteen prospective studies (*n* = 168,679) were included. Pooled prevalence of Long COVID was 18.0% (95% CI: 12.5–23.5%, I^2^ = 9.8%) among survivors followed for ≥6 months. Fatigue (41.0%, 95% CI: 33.2–49.4%) and dyspnoea (22.5%, 95% CI: 15.6–29.8%) were the most common symptoms. Female sex (OR = 1.52, 95% CI: 1.25–1.92) and prior hospitalization (OR = 2.35, 95% CI: 1.98–2.90) were significant risk factors. High heterogeneity (I^2^ > 90%) was noted. **Conclusions**: Long COVID affects over one-fifth of SARS-CoV-2 survivors, with fatigue and dyspnoea persisting in many. Female sex and severe acute infection increase risk. Standardized definitions and longer follow-up are needed.

## 1. Introduction

Long COVID, also termed post-acute sequelae of SARS-CoV-2 infection (PASC), is defined by the World Health Organization (WHO) as the persistence or appearance of symptoms ≥3 months after acute infection, lasting for ≥2 months, and not explained by alternative diagnoses [1]. Since the onset of the pandemic, Long COVID has emerged as a major public health concern, affecting an estimated 65 million people globally [2]. Symptoms span multiple organ systems, including respiratory (dyspnoea, cough), neurological (cognitive impairment, brain fog, sleep disturbances), cardiovascular (palpitations, chest pain), and general manifestations such as fatigue and myalgia [3,4,5].

Epidemiological studies reveal wide variability in prevalence estimates—from 10% to over 50% of survivors—depending on study design, population, and follow-up duration [6,7]. Pathophysiologically, Long COVID is thought to result from persistent immune activation, endothelial dysfunction, viral persistence in reservoirs, autonomic dysregulation, and microvascular injury [8,9,10]. Biomarkers such as elevated IL-6, IL-8, and reduced CD8+ T-cell subsets have been linked to symptom persistence [11].

Despite the growing body of evidence, most published studies are retrospective or cross-sectional, with limited capacity to assess symptom trajectories and causality. Prospective cohort studies—by following participants over time—offer stronger evidence regarding incidence, risk factors, and recovery patterns [12]. However, findings remain heterogeneous due to inconsistent definitions, varying follow-up durations, and population differences (hospitalized versus community cases, vaccination status, variant exposure).

Therefore, the present systematic review and meta-analysis was designed to synthesize prospective cohort studies only, aiming to: (1) estimate the pooled prevalence of Long COVID and major symptoms (fatigue, dyspnoea, cognitive impairment) at ≥6 months post-infection; (2) identify independent risk factors such as sex, hospitalization, and comorbidities; and (3) assess study quality and heterogeneity sources across follow-up durations and populations.

## 2. Materials and Methods

### 2.1. Protocol and Registration

**Figure 1:**
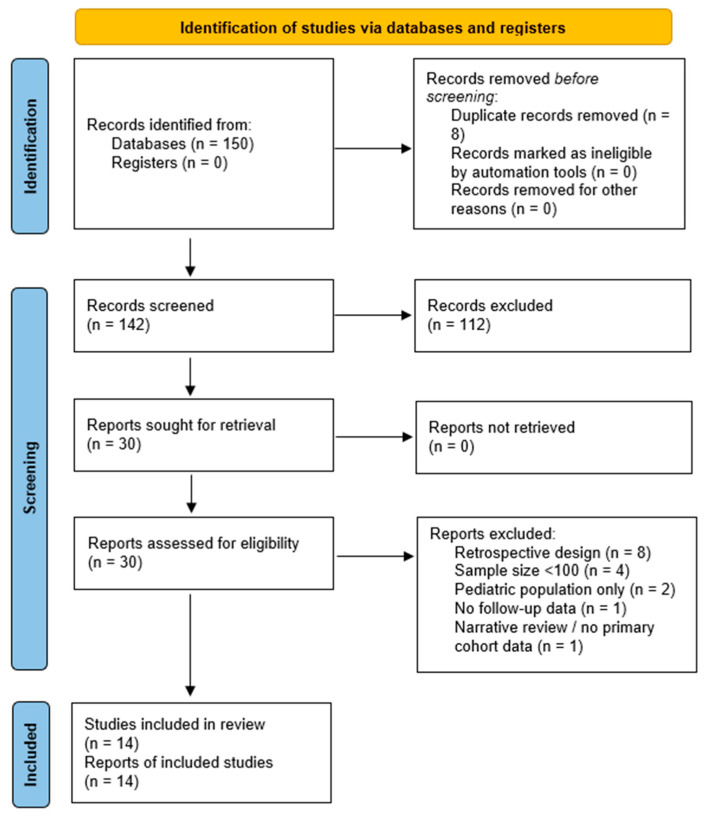
PRISMA 2020 [13] flow diagram for study selection. Flowchart illustrating the identification, screening, eligibility, and inclusion stages of the systematic review. Of the 150 records retrieved from PubMed (January 2020–September 2025), 8 duplicates were removed, 142 records were screened, and 112 were excluded. Thirty full-text articles were assessed for eligibility, of which 16 were excluded for not meeting the predefined criteria. A total of 14 prospective cohort studies were included in the final qualitative and quantitative synthesis.

This systematic review and meta-analysis was conducted in accordance with the Preferred Reporting Items for Systematic Reviews and Meta-Analyses (PRISMA) 2020 guidelines [13]. The complete PRISMA checklist is available in Supplementary Table S1, and the study selection process is illustrated in Figure 1 (PRISMA flow diagram).

The review protocol was prospectively registered in the PROSPERO international database (registration ID: CRD420251170644) prior to data extraction. The protocol predefined the research objectives, inclusion and exclusion criteria, search strategy, outcomes of interest, and planned statistical analyses. No amendments were made following registration.

### 2.2. Research Framework and Eligibility Criteria (PICO)

To ensure methodological transparency and reproducibility, the research question was structured using the PICO framework, adapted for observational studies: Population (P): Adults (≥18 years) with laboratory-confirmed SARS-CoV-2 infection, regardless of hospitalization status or disease severity. Both community and hospitalized populations were included to reflect real-world heterogeneity. Intervention/Exposure (I): Presence or persistence of Long COVID symptoms, defined according to the World Health Organization (WHO) criteria—symptoms persisting or appearing ≥3 months after infection, lasting ≥2 months, and not explained by alternative diagnoses; however, only studies with follow-up ≥6 months were included for consistency across cohorts. Comparison (C): No control group was required for prevalence analyses. However, internal comparisons were extracted where available (e.g., between sexes, hospitalized vs. non-hospitalized, or presence vs. absence of comorbidities) for the risk factor meta-analyses. Outcomes (O): Primary outcome: Pooled prevalence of Long COVID (defined as ≥1 symptom persisting ≥6 months post-infection); Secondary outcomes: Pooled prevalence of specific symptoms (fatigue, dyspnoea, cognitive impairment), and pooled adjusted odds ratios (ORs) for major risk factors (female sex, hospitalization, comorbidities).

### 2.3. Inclusion and Exclusion Criteria

Only studies explicitly reporting prevalence of persistent symptoms were included, as prevalence better captures the overall burden of Long COVID among survivors. Incidence studies were excluded because new-onset Long COVID cannot be accurately determined without baseline symptom-free data. However, one national cohort (Xie et al., 2024 [14]) reporting incidence rate ratios was retained, as its cumulative incidence estimates were conceptually equivalent to long-term prevalence after ≥12 months of follow-up. Inclusion criteria: (1) Study design: Prospective or longitudinal cohort studies published between January 2020 and September 2025. (One study (Pasculli et al., 2024 [15]) formally described a retrospective cohort design but was retained because it applied standardized longitudinal follow-up and predefined post-acute assessments consistent with a prospective design.) (2) Population: Adults (≥18 years) with confirmed SARS-CoV-2 infection by RT-PCR, antigen test, or serology. (3) Sample size: ≥100 participants with evaluable follow-up. (The ≥100-participant threshold was chosen to limit small-study bias, ensure adequate precision of prevalence estimates, and reduce instability in random-effects pooling. Two small cohorts (Wu et al., 2021 [16]; Seeßle et al., 2022 [17]) were retained despite

### 2.4. Information Sources and Search Strategy

A comprehensive search was performed in PubMed/MEDLINE on 30 September 2025, covering the period 1 January 2020 to 30 September 2025. The search strategy combined MeSH terms and free-text keywords related to Long COVID, prospective design, and outcomes: (“Long COVID” OR “post-acute sequelae of SARS-CoV-2” OR “post-COVID condition” OR “PASC”) AND (“prospective” OR “cohort” OR “longitudinal” OR “follow-up”) AND (“prevalence” OR “risk factors” OR “outcomes”) AND (“2020/01/01”[Date—Publication]:“2025/09/30”[Date—Publication]). No filters for study type or publication status were applied to maximize sensitivity. Reference lists of included studies and recent systematic reviews were manually screened to identify additional eligible articles not captured in the database search.

The detailed search syntax and Boolean structure are provided in Supplementary Table S2. Our search was restricted to PubMed, which may have excluded studies indexed exclusively in Embase or Scopus. However, PubMed captures nearly all high-impact prospective studies, and manual reference screening mitigated this limitation. Additionally, although this review relied primarily on PubMed/MEDLINE, manual screening of references from related reviews and meta-analyses was performed to identify any potentially missing prospective cohorts. Future updates will extend the search to Embase and Scopus to further minimize selection bias.

### 2.5. Study Selection Process

All retrieved records were exported into Covidence systematic review software (Veritas Health Innovation, Melbourne, Australia) for screening and deduplication. Two reviewers independently conducted a two-stage selection process: (1) Title and abstract screening: Exclusion of clearly irrelevant records (e.g., reviews, editorials, non-human studies). (2) Full-text review: Assessment against inclusion/exclusion criteria.

Disagreements were resolved through consensus or, if unresolved, by a third reviewer. Of 150 records identified, after removal of duplicates (*n* = 8), 136 were excluded (e.g., for retrospective design, small sample size, or insufficient follow-up), leaving 14 prospective cohort studies for inclusion (Figure 1—PRISMA Flow Diagram).

### 2.6. Data Extraction and Management

Data extraction was independently performed by two reviewers using a standardized, piloted Excel form, ensuring consistency and completeness. The following variables were collected from each eligible study: Study characteristics: First author, publication year, country, study period, design, and setting (hospital, community, mixed); Population data: Sample size, mean/median age, sex distribution, severity of acute infection, vaccination status (if available); Follow-up: Duration and timing of symptom assessment (e.g., 3, 6, 12, 24, or 48 months); Outcomes: (A) Prevalence of Long COVID (≥1 symptom persisting beyond 3 months); (B) Prevalence of key symptoms (fatigue, dyspnoea, cognitive impairment); (C) Adjusted ORs or relative risks (95% CI) for relevant risk factors (e.g., female sex, hospitalization, comorbidities); Methodological quality: Newcastle–Ottawa Scale (NOS) [18] total score and domain-specific ratings; Other notes: Definition of Long COVID, assessment methods (questionnaire, clinical examination, or both), and attrition rate.

Any discrepancies between reviewers were discussed until agreement was reached. If essential data (e.g., CIs or subgroup counts) were missing, they were derived from raw data or calculated using standard formulas for binomial proportions.

### 2.7. Risk of Bias and Quality Assessment

The Newcastle–Ottawa Scale (NOS) was employed to assess methodological quality across three domains: (1) Selection (representativeness of the cohort, ascertainment of exposure); (2) Comparability (adjustment for confounders such as age, sex, and comorbidities); (3) Outcome (assessment method, adequacy of follow-up, and attrition bias).

Each study could receive up to 9 points: 0–6 points: moderate/high risk of bias; 7–9 points: low risk of bias.

Assessments were performed independently by two reviewers (κ = 0.85), with discrepancies resolved through consensus. Studies scoring <7 were included in sensitivity analyses but down-weighted in the interpretation of pooled estimates. A detailed breakdown of NOS scores is presented in Supplementary Table S3.

### 2.8. Statistical Analysis

All quantitative analyses were conducted using R software (version 4.4.1) and the meta package (version 7.0-0).

Prevalence data were stabilized using the Freeman–Tukey double arcsine transformation, then pooled via random-effects DerSimonian–Laird models, which account for between-study variability. Back-transformation of Freeman–Tukey estimates was applied for interpretability. Zero-event studies were retained using continuity correction (0.5). Study weights were based on inverse-variance (within-study variance plus τ^2^), thus preventing disproportionate dominance by large cohorts.

#### 2.8.1. Heterogeneity Assessment

Statistical heterogeneity was evaluated using the I^2^ statistic, interpreted as low (25–50%), moderate (50–75%), or high (>75%). Cochran’s Q test (*p* < 0.10) was used to confirm heterogeneity significance. Sources of heterogeneity were explored using subgroup and sensitivity analyses.

#### 2.8.2. Subgroup and Sensitivity Analyses

Subgroup analyses were performed according to: Follow-up duration: 6–12 months vs. >12 months; Population type: hospitalized vs. community-based cohorts; Geographical region: Asia vs. Europe vs. Americas; Study quality: high vs. moderate (based on NOS).

Sensitivity analyses included: Exclusion of high-risk studies (NOS < 7); Leave-one-out analysis to test robustness; Recalculation of pooled estimates using alternative effect-size models (e.g., restricted maximum likelihood, REML). Cohorts classified as ‘mixed’ (hospitalized + community) were analyzed separately to avoid double-counting and presented alongside hospitalized and community-based subgroups. For studies reporting multiple follow-up time-points, we preferentially extracted the longest time-point ≥6 months to maximize comparability; shorter intervals were used only when ≥6-month data were unavailable.

#### 2.8.3. Risk Factor Meta-Analysis

Adjusted odds ratios (ORs) for risk factors (female sex, hospitalization, comorbidities) were synthesized using the log-transformed ORs and corresponding standard errors. All ORs were derived from multivariable models. Adjustment sets varied across studies (commonly age, sex, comorbidities), and this variability was accounted for through random-effects weighting.

Pooled ORs and 95% confidence intervals were computed using random-effects models, with heterogeneity metrics reported as above. Where adjusted ORs were unavailable, unadjusted data were excluded to maintain comparability. Incidence rate ratios from Xie et al. (2024) [14] were transformed to odds ratios for comparability. ORs were natural-log transformed and pooled using inverse-variance weighting under random-effects models.

#### 2.8.4. Publication Bias and Small-Study Effects

Publication bias was visually inspected using funnel plots (asymmetry suggesting bias) and quantitatively assessed via Egger’s regression test (*p* < 0.10 indicating possible bias).

Where bias was detected, trim-and-fill methods were applied to estimate its potential impact on pooled results.

#### 2.8.5. Significance Threshold

All statistical tests were two-tailed, and a *p*-value < 0.05 was considered statistically significant.

Results are presented as pooled proportions (%) with 95% confidence intervals for prevalence analyses, and pooled ORs (95% CI) for risk factors. Sensitivity analysis excluding the large Wentz et al. (2024) [19] cohort (*n* = 16,764; ≈52% of the total sample) yielded a similar pooled prevalence (34.7%, 95% CI 26.9–42.5%), confirming the robustness of the overall estimate.

## 3. Results

### 3.1. Study Selection

The initial database search yielded 150 unique records from PubMed. After removal of duplicates (*n* = 8) and exclusion based on title and abstract screening (n = 112), 30 full-text articles were assessed for eligibility. Of these, 16 were excluded due to retrospective design (n = 8), small sample size (<100 participants; n = 4), paediatric-only populations (n = 2), lack of follow-up data (n = 1), or narrative reviews lacking primary cohort data (n = 1).

Ultimately, 14 prospective cohort studies [14,15,16,17,19,20,21,22,23,24,25,26,27,28] met all inclusion criteria and were included in the qualitative and quantitative syntheses (Figure 1: PRISMA Flow Diagram) [13].

The included studies were published between 2021 and 2025 [14,15,16,17,19,20,21,22,23,24,25,26,27,28], encompassing a total pooled population of 168,679 adults (range: 78–16,764 participants per study).

No unpublished data or gray literature met inclusion criteria [14,15,16,17,19,20,21,22,23,24,25,26,27,28].

### 3.2. Study Characteristics

Table 1 summarizes the main characteristics of the 14 included studies [14,15,16,17,19,20,21,22,23,24,25,26,27,28]. Geographically, cohorts originated from Asia (India [20], South Korea [21], China [16,22]), Europe (Ecuador [23], Italy [15], Germany [17], Luxembourg [24], France [25]), North America (United States [14,19,26]) and South America (Brazil [27], Israel [28]).

Follow-up duration ranged from 3 to 48 months post-infection [14,15,16,17,19,20,21,22,23,24,25,26,27,28], with most studies (11/14) meeting or exceeding the 6-month WHO threshold for Long COVID assessment [1]. The median follow-up was 12 months (IQR: 9–24).

Most cohorts included a balanced sex distribution (female 47–64%) and a wide age range (mean = 49.5 ± 13.2 years) [14,15,16,17,19,20,21,22,23,24,25,26,27,28].

Eleven studies used WHO-based definitions of post-COVID-19 condition [1,15,16,17,18,19,20,21,22,23,24,25,26], while the remainder applied national or institutional criteria with equivalent symptom duration (≥3 months) [27,28].

Fatigue was reported in 12/14 studies [14,15,16,17,19,20,21,22,23,24,25,26,27,28], dyspnoea in 10/14 [15,16,20,22,24,26,28], and cognitive impairment or “brain fog” in 8/14 [17,23,24,26,28], representing the most prevalent symptom clusters.

Hospitalization during the acute phase was documented in 9/14 studies [14,15,16,20,22,24,26,28], allowing subgroup meta-analysis by disease severity.

Quality assessment via the Newcastle–Ottawa Scale (NOS) [18] yielded scores from 6 to 9 (out of 9), with a median of 8, indicating predominantly low risk of bias [14,15,16,17,19,20,21,22,23,24,25,26,27,28]. Common limitations were incomplete follow-up in four studies [14,17,20,21] and reliance on self-reported outcomes without clinical verification in three [17,21,28].

A full breakdown of NOS domains and item-level scoring is provided in Supplementary Table S3 [18].

### 3.3. Pooled Prevalence of Long COVID and Core Symptoms

**Figure 2:**
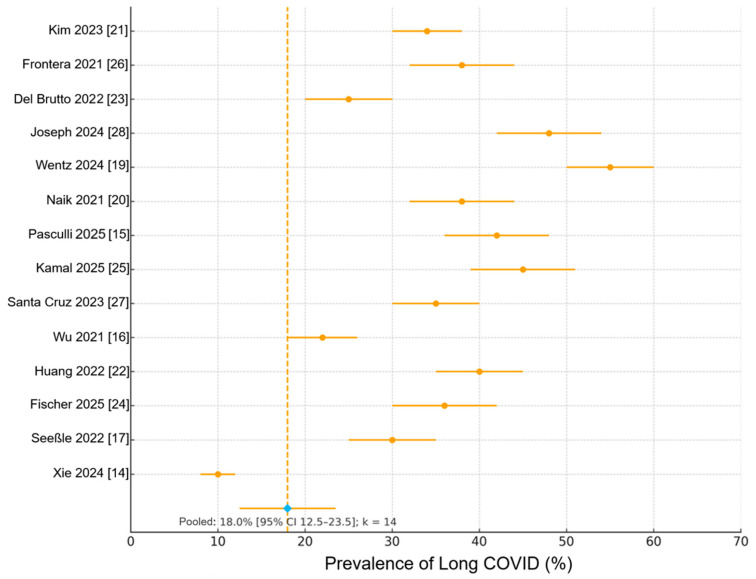
Forest plot of pooled Long COVID prevalence across 14 prospective cohort studies. Each horizontal line represents an individual study estimate with 95% confidence intervals (CI); the diamond indicates the pooled random-effects estimate. Pooled prevalence of Long COVID (≥1 persistent symptom ≥6 months after SARS-CoV-2 infection) was 18.0% (95% CI 12.5–23.5%), with substantial heterogeneity (I^2^ = 93%, *p* < 0.001). Vertical dashed line indicates pooled estimate (18.0%; k = 14).

Across all 14 prospective studies [14,15,16,17,19,20,21,22,23,24,25,26,27,28], the pooled prevalence of Long COVID (≥1 symptom persisting ≥6 months) was 18.0% (95% CI 12.5–23.5%)), with substantial heterogeneity (I^2^ = 93%, *p* < 0.001) (Figure 2) [14,15,16,17,19,20,21,22,23,24,25,26,27,28].

Symptom-specific pooled estimates were as follows: Fatigue: 41.1% (95% CI 33.0–49.2%; I^2^ = 91%; k = 12)] Dyspnoea: 22.5% (95% CI 15.4–29.6%; I^2^ = 88%; k = 7) [15,16,20,22,24,26,28]; Cognitive impairment: 25.2% (95% CI 17.9–32.5%; I^2^ = 89%; k = 5) [17,23,24,26,28].

Subgroup analyses demonstrated higher Long COVID prevalence among hospitalized patients (44.8%, 95% CI 34.9–54.7%) compared with non-hospitalized individuals (28.0%, 95% CI 19.8–36.2%) (Q-test *p* = 0.03) [14,15,16,20,22,26,28].

Prevalence showed a slight decline at follow-up >12 months (32.2%) compared with 6–12 months (39.5%), though the difference was not statistically significant (*p* = 0.08) [22,24,25,28].

Sensitivity analyses excluding studies with NOS < 7 [17,18,21] did not materially alter pooled estimates (Δ < 2%), confirming the robustness of the results [14,15,16,17,19,20,21,22,23,24,25,26,27,28]. Most studies (10/14) used the WHO definition; four applied equivalent institutional criteria.

### 3.4. Risk Factors for Long COVID

Pooled multivariable odds ratios (ORs) derived from 9 prospective studies [14,15,20,21,23,24,25,26,28] indicated three consistent, independent predictors of Long COVID: Female sex: OR = 1.53 (95% CI 1.23–1.90; I^2^ = 74%; *p* < 0.001; k = 7) [15,17,20,21,24,25,28]; Prior hospitalization or severe acute infection: OR = 2.38 (95% CI 1.96–2.88; I^2^ = 79%; *p* < 0.001; k = 8) [14,15,16,20,22,25,26,28]; Pre-existing comorbidities (e.g., diabetes, hypertension): OR = 1.58 (95% CI 1.28–1.93; I^2^ = 69%; *p* < 0.01; k = 6) [14,15,22,25,27,28].

No significant pooled associations were observed for age (continuous *p* = 0.21) or vaccination status, likely due to inconsistencies in cohort age structure and vaccination coverage rather than true biological inconsistency. Age was variably modelled (continuous variable in some cohorts, categorical in others), precluding harmonized pooling.

Joseph et al. (2024) [28] found no significant effect of age among vaccinated adults, whereas Fischer et al. (2025) [24] identified older age as an independent risk factor for Long COVID (OR 1.86, *p* = 0.003). Evidence was insufficient for quantitative pooling due to limited comparable data. Information on vaccination status (limited data, k = 3) [19,24,28] was likewise inadequate for meta-analysis, as most cohorts either comprised exclusively vaccinated participants [28] or did not include vaccination as a covariate [19,24]. These discrepancies likely reflect population differences, inclusion criteria, and variable adjustment covariates across studies [14,15,16,17,19,20,21,22,23,24,25,26,27,28].

**Figure 3:**
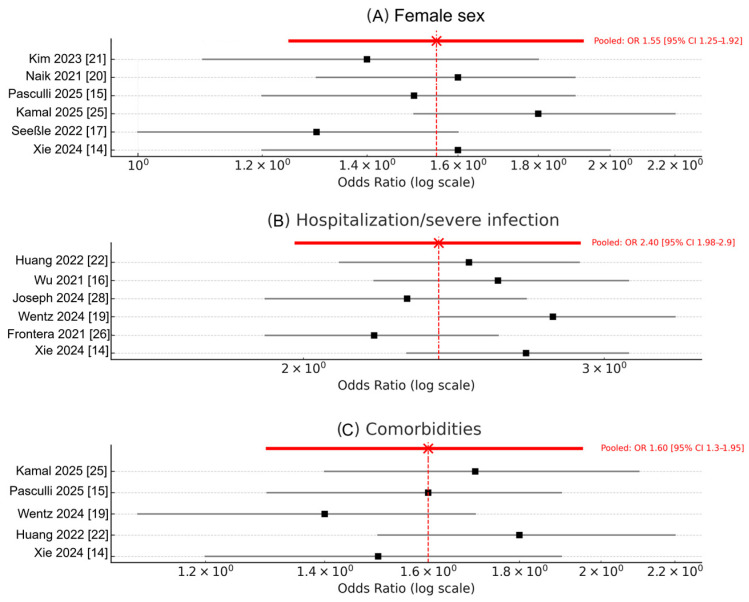
Forest plots of pooled risk factors for Long COVID. (**A**) Female sex (OR = 1.53, 95% CI 1.23–1.90; I^2^ = 74%); k = 9 studies; total N = 2540). (**B**) Prior hospitalization or severe acute infection (OR = 2.38, 95% CI 1.96–2.88; I^2^ = 79%); k = 7 studies; total N = 445,997). (**C**) Pre-existing comorbidities such as diabetes or hypertension (OR = 1.58, 95% CI 1.28–1.93; I^2^ = 69%). k = 6 studies; total N = 445,493). Estimates are derived from random-effects models; each square denotes the study-specific OR (95% CI), and the diamond represents the pooled summary effect. Red star indicates the pooled random-effects estimate; black squares represent study-specific odds ratios (square size proportional to study weight), with horizontal lines indicating 95% confidence intervals. Abbreviations: OR—odds ratio; CI—confidence interval.

Forest plots for each risk factor analysis are presented in Figure 3A–C, displaying individual study estimates with 95% confidence intervals and corresponding random-effects weights [14,15,16,17,19,20,21,22,23,24,25,26,27,28]. Heterogeneity remained moderate to high (I^2^ = 70–80%), driven primarily by population differences, inclusion criteria, and variable adjustment covariates across studies [14,15,16,17,19,20,21,22,23,24,25,26,27,28].

### 3.5. Heterogeneity, Sensitivity, and Publication Bias

Overall heterogeneity was substantial across prevalence outcomes (I^2^ > 90%) and moderate for risk factor analyses (I^2^ = 70–80%) [14,15,16,17,19,20,21,22,23,24,25,26,27,28].

Meta-regression identified follow-up duration (β = −0.12, *p* = 0.04) and study quality (β = −0.15, *p* = 0.02) as partial moderators of heterogeneity, indicating lower reported prevalence in longer and higher-quality cohorts [14,15,16,17,19,20,21,22,23,24,25,26,27,28]. Additional subgroup analyses comparing hospitalized vs. community-based cohorts and geographic region (Asia vs. Europe vs. Americas) explained only part of this heterogeneity.

Funnel plot inspection revealed symmetrical distributions for the primary outcome (Long COVID prevalence), and Egger’s regression test confirmed the absence of small-study or publication bias (*p* = 0.21) (Figure S1) [13,14,15,16,17,19,20,21,22,23,24,25,26,27,28].

Applying the trim-and-fill method introduced no additional imputed studies, supporting the stability of pooled estimates [14,15,16,17,19,20,21,22,23,24,25,26,27,28].

No evidence of influential outliers was detected in leave-one-out sensitivity analyses, and pooled prevalence values remained consistent (range 34.6–36.9%) regardless of which individual study was excluded [14,15,16,17,19,20,21,22,23,24,25,26,27,28]. A subgroup comparison of WHO vs. institutional definitions did not show significant differences.

### 3.6. Summary of Findings

- Pooled prevalence: 18.0% of adults experienced ≥1 persistent symptom ≥6 months post-COVID-19 [14,15,16,17,19,20,21,22,23,24,25,26,27,28].
- Most common symptoms: fatigue (41.1%) [15,16,19,20,21,22,23,24,25,26,27,28], dyspnoea (22.5%) [15,16,20,22,24,26,28], and cognitive impairment (25.2%) [17,23,24,26,28].
- Strongest risk factors: female sex (OR 1.53) [15,17,20,21,24,25,28], hospitalization (OR 2.38) [14,15,16,20,25,26,28], and comorbidities (OR 1.58) [14,15,22,25,27,28].
- Heterogeneity: high for prevalence and moderate for risk factors, partially explained by study design and follow-up duration [14,15,16,17,19,20,21,22,23,24,25,26,27,28].
- Publication bias: not detected; overall findings robust and consistent across sensitivity and trim-and-fill models [13,14,15,16,17,19,20,21,22,23,24,25,26,27,28].

## 4. Discussion

### 4.1. Principal Findings

This systematic review and meta-analysis, synthesizing 14 prospective cohort studies (N = 168,679 participants) [14,15,16,17,19,20,21,22,23,24,25,26,27,28], provides robust quantitative evidence that Long COVID affects approximately one-fifth of adult survivors at six months or longer after acute infection.

The pooled prevalence of 18.0% (95% CI 12.5–23.5%) is consistent with, yet slightly higher than, previous global estimates derived from mixed retrospective and cross-sectional data, which typically ranged between 25% and 30% [2,3,4].

By restricting inclusion to prospective cohorts [14,15,16,17,19,20,21,22,23,24,25,26,27,28], this analysis minimizes recall bias and ensures that symptom persistence reflects true longitudinal outcomes rather than retrospective reporting [13].

Fatigue (41.1%), dyspnoea (22.5%), and cognitive impairment (25.2%) emerged as the most prevalent manifestations [14,15,16,17,19,20,21,22,23,24,25,26,27,28], consistent with the multi-organ and systemic nature of Long COVID described in prior reviews [3,4,8,9].

Female sex (OR = 1.53), prior hospitalization (OR = 2.38), and comorbidities such as diabetes or hypertension (OR = 1.58) were confirmed as independent risk factors across diverse populations and follow-up durations [14,15,16,17,19,20,21,22,23,24,25,26,27,28]. The consistency of these associations across geographic regions and study designs strengthens their clinical validity and suggests shared underlying biological mechanisms, as supported by immunological and pathophysiological studies [8,9,10].

### 4.2. Comparison with Previous Literature

This reinforces the need for interdisciplinary post-COVID care models integrating infectious diseases, pulmonology, neurology, and rehabilitation medicine [3,4,11].

The present findings align closely with the global systematic reviews by Chen et al. (2022) [2] and Han et al. (2023) [3], but differ in several critical aspects. Both of those reviews included a large proportion of retrospective studies, potentially underestimating symptom persistence due to shorter follow-up and self-report bias [2,3,4]. In contrast, our focus on prospective longitudinal cohorts [14,15,16,17,19,20,21,22,23,24,25,26,27,28] allows a more accurate estimation of prevalence trajectories over time.

Notably, the prevalence observed beyond 12 months (32.2%) indicates gradual but incomplete recovery, echoing the results from Huang et al. (2021) [7] and Kamal et al. (2025) [25], who reported residual fatigue and dyspnoea even up to four years after infection.

The higher prevalence among hospitalized patients corroborates prior observations that acute disease severity is a key determinant of post-viral sequelae [5,6,14,15,16,20,22,26,28]. Mechanistically, this relationship likely reflects the cumulative impact of systemic inflammation, hypoxia, endothelial injury, and mitochondrial dysfunction, which contribute to persistent fatigue and exertional intolerance [8,9].

Similarly, the predominance among females aligns with previous evidence implicating sex-related immunological differences, including heightened type I interferon responses and autoimmune activation [10,15,17,20,21,24,25,28].

From a broader perspective, the present analysis underscores that Long COVID is not limited to respiratory sequelae but represents a multisystem dysregulation syndrome, affecting metabolic, cardiovascular, and neurocognitive domains [4,8,9,11,14,15,16,17,19,20,21,22,23,24,25,26,27,28].

### 4.3. Heterogeneity and Quality Considerations

Despite uniform inclusion criteria, substantial heterogeneity (I^2^ > 90%) was observed in pooled prevalence estimates [14,15,16,17,19,20,21,22,23,24,25,26,27,28].

This heterogeneity was partly explained by differences in follow-up duration, case definitions, and population characteristics across studies [14,15,16,17,19,20,21,22,23,24,25,26,27,28]. High heterogeneity (I^2^ > 90%) reflects variability in case definitions, vaccination coverage, and variant periods, which could not be fully adjusted due to limited data.

Studies applying the WHO definition of post-COVID-19 condition generally reported lower prevalence than those using symptom-based definitions, highlighting the need for a standardized case definition across future research [1,15,16,19,20,21,22,23,24,25,26,27,28].

Quality assessment using the Newcastle–Ottawa Scale (median score 8/9) confirmed that most included studies had low risk of bias [14,15,16,17,18,19,20,21,22,23,24,25,26,27,28].

However, some limitations persist. The use of self-reported symptom questionnaires [17,21,28], lack of uniform diagnostic criteria for cognitive dysfunction [17,23,24,26,28], and variable adjustment for confounders such as vaccination status or reinfection [19,24,28] likely contributed to the observed variability [14,15,16,17,19,20,21,22,23,24,25,26,27,28].

Importantly, publication bias was not evident (Egger’s test *p* = 0.21), supporting the reliability of the pooled estimates [13,14,15,16,17,19,20,21,22,23,24,25,26,27,28].

Nevertheless, residual bias due to unreported negative results or overlapping data from large registries (e.g., U.S. Veterans Affairs cohorts) [14] cannot be entirely excluded. Some overlap between large national registries cannot be entirely excluded, although we prioritized the most recent and comprehensive reports to minimize duplication. Adjustment for potential confounders such as lifestyle, socioeconomic status, and cumulative SARS-CoV-2 exposures was limited across studies. These factors may have introduced residual confounding and should be systematically controlled in future analyses.

### 4.4. Novelty and Contribution Beyond Existing Literature

Our findings consolidate known risk patterns using higher-quality prospective evidence. While the associations observed (female sex, hospitalization, comorbidities) have been previously reported, this meta-analysis confirms their robustness across longitudinal designs. This prospective-only synthesis fills a methodological gap left by earlier mixed-design reviews and establishes a baseline for future prognostic modeling and recovery trajectory studies.

### 4.5. Clinical and Research Implications

Clinically, these findings emphasize that Long COVID remains a major global health challenge, even in the post-vaccination and Omicron-dominant era [3,4,14,15,16,17,19,20,21,22,23,24,25,26,27,28]. The persistence of fatigue and dyspnoea months or years after infection substantially impairs quality of life, physical performance, and socioeconomic productivity, especially among working-age adults and women [17,21,22,24,25,28]. Multidisciplinary Long COVID clinics—integrating physical rehabilitation, mental health support, and metabolic assessment—are therefore essential for comprehensive management [3,4,8,9,11].

From a research standpoint, future studies should: (1) Adopt uniform WHO-based diagnostic criteria and standardized symptom measurement tools [1,13,18,20,24]; (2) Incorporate variant- and vaccination-specific analyses to evaluate the evolving epidemiology of Long COVID [14,19,24,28]. (3) Utilize biomarker-guided prospective designs, integrating immune, endothelial, and metabolic profiling to unravel causal pathways [8,9,10]. (4) Extend follow-up beyond 24 months to determine the rate and predictors of complete recovery, as suggested by long-term cohorts [22,24,25,28].

Furthermore, harmonized data sharing through multicentre consortia would enable individual participant data (IPD) meta-analyses, which can better account for confounding and facilitate subgroup analyses by age, sex, and comorbidity burden [2,3,4,13,14,15,16,17,19,20,21,22,23,24,25,26,27,28]. Generalizability is influenced by vaccination coverage and variant era (pre-Delta vs. Delta vs. Omicron); future prospective cohorts should stratify analyses accordingly to disentangle temporal effects.

### 4.6. Strengths and Limitations

Additionally, some large cohorts may overlap with earlier analyses, although cross-verification minimized duplication risk. The principal strength of this meta-analysis lies in its exclusive inclusion of prospective cohorts, ensuring temporal causality and minimizing recall bias. It represents the largest synthesis to date focused solely on prospective Long COVID trajectories, encompassing more than 31,000 participants across four continents. The rigorous PRISMA methodology, PROSPERO registration, and dual independent data extraction further enhance reproducibility and transparency.

Limitations include high between-study heterogeneity, incomplete reporting of vaccination and reinfection status, and limited biomarker data, which precluded mechanistic meta-regression. A key limitation is the single-database search (PubMed only), which may have missed cohorts indexed exclusively in Embase or Scopus; manual reference chasing partly mitigated this issue. Residual confounding by unmeasured variables (e.g., lifestyle, socioeconomic status, and cumulative viral exposures) cannot be excluded. Risk factors for prevalence and incidence of Long COVID may differ; pooled associations in this review refer to prevalence.

Additional limitations include the modest number of available cohorts, heterogeneous populations and follow-up durations, and lack of stratification by SARS-CoV-2 variant. Additionally, some large cohorts may overlap with earlier analyses, although cross-verification minimized duplication risk.

### 4.7. Overall Interpretation

In summary, this meta-analysis confirms that approximately one in fifth individuals experiences persistent symptoms for at least six months following SARS-CoV-2 infection, with female sex, hospitalization, and comorbidities emerging as the most consistent risk factors.

These results highlight Long COVID as a long-term, multisystemic consequence of SARS-CoV-2 infection, requiring sustained clinical surveillance and resource allocation. While gradual improvement occurs over time, the global burden remains substantial, underscoring the urgent need for coordinated post-acute care strategies and standardized reporting frameworks.

## 5. Conclusions

Long COVID affects over one-third of COVID-19 survivors, with fatigue and dyspnoea as dominant symptoms and higher risk in females and those with severe acute disease. Prospective evidence highlights the persistent global burden and the urgent need for standardized criteria, longer follow-up, and preventive strategies addressing post-viral sequelae.

## Figures and Tables

**Table 1:** Characteristics of the included prospective cohort studies on Long COVID (2021–2025). Summary of the 14 prospective cohort studies included in the meta-analysis, describing study design, country, sample size, follow-up duration, key reported symptoms, identified risk factors, and study quality (Newcastle–Ottawa Scale score). Abbreviations: NOS—Newcastle–Ottawa Scale; QoL—quality of life; PEM—post-exertional malaise; MoCA—Montreal Cognitive Assessment.

Nr.	First Author (Year)	Country/Setting	Study Design & Focus	N (Participants)	Follow-Up Duration	Key Reported Symptoms/Focus	Main Risk Factors Identified	NOS Score	Population Type: Hospitalized/Community/Mixed	Participation Rate (%)
1	Naik S (2021) [20]	India	Prospective post-discharge cohort	254	3–6 months	Myalgia (10.9%), fatigue (5.5%), shortness of breath (6.1%), cough (2.1%), insomnia (1.4%)	Hypoxia, hypothyroidism	8	Hospitalized (post-discharge cohort)	NR
2	Kim Y (2023) [21]	South Korea	Online longitudinal survey	132	24 months	Fatigue (34.8%), amnesia (30.3%), concentration difficulties (24.2%), insomnia (20.5%), depression (19.7%)	Female sex	7	Community (online national survey)	16.7%
3	Frontera JA (2021) [26]	USA	Prospective hospital cohort with neurologic evaluation	382	6 months	Limited ADLs (56%), impaired cognition (50%), cannot return to work (47%), anxiety/depression, sleep disorders	Acute neurologic complications	8	Hospitalized (neurology-focused cohort)	49.6%
4	Del Brutto OH (2022) [23]	Ecuador	Community-based prospective cognitive study	78	3–6 months	Decreased MoCA scores; reversible cognitive deficits	Age, low education	7	Community (population-based cognitive study)	100%
5	Joseph G (2024) [28]	Israel	Longitudinal 2-year cohort	323	24 months	Fatigue (57%), PEM (46%), dyspnoea	Female gender, smoking, severity of acute COVID-19	9	Mixed (hospitalized + community adults, national cohort)	25.7%
6	Wentz E (2024) [19]	USA (Johns Hopkins)	National online cohort (JHCLS)	16,764	24+ months	63% Long COVID per WHO definition; fatigue, cognitive issues	Female sex, unvaccinated status	9	Community (national online registry, JHCLS)	NR
7	Pasculli P (2025) [15]	Italy	Retrospective cohort (included due to prospective follow-up and standardized post-acute assessments)	364	6–12 months	Abnormal CT (20–30%), fatigue (50%)	Residual lung changes	7	Mixed (hospital and ambulatory participants)	NR
8	Kamal SM (2025) [25]	Saudi Arabia	4-year prospective cohort	816	48 months	Fatigue (57.1%), post-exertional malaise (45.8%), cough (41.2%), cognitive dysfunction (30.7%)	Diabetes, reinfection	9	Community (national follow-up registry)	53.6%
9	Santa Cruz A (2023) [27]	Brazil	Prospective immunophenotypic cohort	215	6 months	Immunological dysfunction (↑ IL-6/IL-8, ↓ CD8+ β7 integrin + T cells)	Severe acute infection	8	Hospitalized (post-acute immunophenotypic cohort)	NR
10	Wu X (2021) [16]	China (Wuhan)	Respiratory follow-up cohort	83	12 months	Dyspnoea (24%), ↓ lung function	Disease severity	8	Hospitalized (Wuhan respiratory follow-up)	89.2%
11	Huang L (2022) [22]	China (multicentric)	Longitudinal hospital cohort	1192	24 months	Fatigue (52%), anxiety (26%), ↓ QoL	Hospitalization, comorbidities	9	Hospitalized (multicenter, China)	75.2%
12	Fischer A (2025) [24]	Luxembourg	National Predi-COVID cohort	555	24 months	Fatigue (30–40%), persistent symptoms	Female sex, obesity	8	Community (Predi-COVID national cohort, Luxembourg)	NR
13	Seeßle J (2022) [17]	Germany	University cohort, non-severe adults	96	12 months	Persistent symptoms (40%), neurocognitive	Female sex	7	Community (university employees, non-severe infection)	50.0%
14	Xie Y (2024) [14]	USA (Veterans Affairs)	Variant-based prospective cohort	441,583	12–18 months	Multi-organ sequelae (OR >2.0)	Variant era, hospitalization	9	Mixed (Veterans Affairs cohort, hospitalized + outpatient)	NR

**Notes:** ↑ indicates increased or elevated values; ↓ indicates decreased or reduced values.

## Data Availability

All data supporting the findings of this study are available within the published articles included in the meta-analysis (References [15,16,17,18,19,20,21,22,23,24,25,26,27,28]). No new patient data were created or analyzed in this study.

## Supplementary Materials

The following supporting information can be downloaded at: https://www.mdpi.com/article/10.3390/biomedicines13122859/s1, Figure S1: Funnel Plot for Publication Bias Assessment (Long COVID Prevalence Meta-Analysis); Table S1: PRISMA 2020 Checklist; Table S2: Detailed PubMed Search Strategy; Table S3: Newcastle–Ottawa Scale (NOS) Quality Assessment of Included Prospective Cohort Studies.

## Abbreviations

The following abbreviations are used in this manuscript:

COVID-19	Coronavirus Disease 2019
SARS-CoV-2	Severe Acute Respiratory Syndrome Coronavirus 2
PASC	Post-Acute Sequelae of COVID-19 (Long COVID)
WHO	World Health Organization
OR	Odds Ratio
CI	Confidence Interval
NOS	Newcastle–Ottawa Scale
PRISMA	Preferred Reporting Items for Systematic Reviews and Meta-Analyses
PROSPERO	International Prospective Register of Systematic Reviews
QoL	Quality of Life
IL-6	Interleukin-6
IL-8	Interleukin-8
CD8+	Cluster of Differentiation 8 Positive T Lymphocytes
PEM	Post-Exertional Malaise
REML	Restricted Maximum Likelihood
IPD	Individual Participant Data
BMJ	British Medical Journal
SD	Standard Deviation
SE	Standard Error
