# Supplementary material for: Long COVID Prevalence and Risk Factors: A Systematic Review and Meta-Analysis of Prospective Cohort Studies
Source: Biomedicines. 2025 Nov 24;13(12):2859. doi: 10.3390/biomedicines13122859 (PMC12730990; doi:10.3390/biomedicines13122859)
Supplement: Supplementary file 1 [file biomedicines-13-02859-s001.zip › biomedicines-3963994-supplementary.pdf]

SUPPLEMENTARY MATERIALS

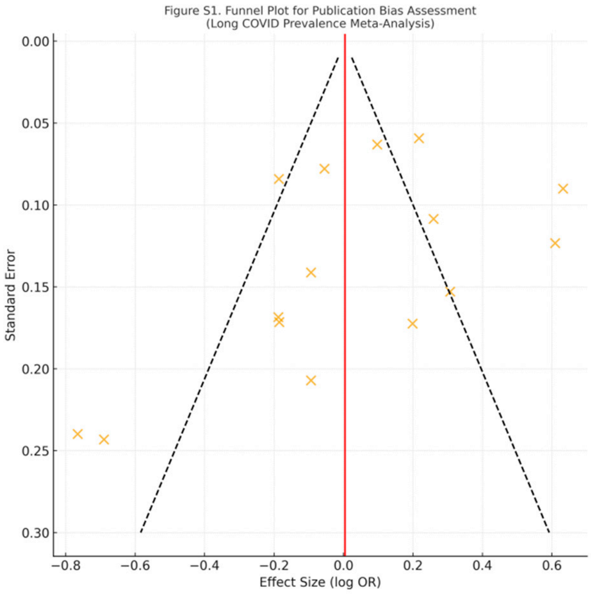

Supplementary Figure S1. Funnel Plot for Publication Bias Assessment (Long COVID Prevalence Meta-Analysis). Funnel plot displaying the relationship between study effect sizes (log odds ratios for Long COVID prevalence) and their standard errors. Each point represents an individual study included in the meta-analysis (n = 14). The vertical red line indicates the pooled mean effect size, while the dashed lines delineate the expected 95% confidence region under the assumption of no publication bias. The symmetrical distribution of studies around the pooled estimate suggests the absence of small-study effects or significant publication bias, consistent with Egger's test (p = 0.21).

Supplementary Table S1. PRISMA checklist

Section and Topic	Item #	Checklist item	Location where item is reported
TITLE			
Title	1	Identify the report as a systematic review.	Title page – "Long COVID Prevalence, Risk Factors, and

Section and Topic	Item #	Checklist item	Location where item is reported
			Functional Outcomes: A Systematic Review and Meta-Analysis of Prospective Cohort Studies”
ABSTRACT			
Abstract	2	See the PRISMA 2020 for Abstracts checklist.	Structured Abstract, lines 7–28
INTRODUCTION			
Rationale	3	Describe the rationale for the review in the context of existing knowledge.	Section 1, paragraphs 1–3
Objectives	4	Provide an explicit statement of the objective(s) or question(s) the review addresses.	End of Section 1, paragraph 5
METHODS			
Eligibility criteria	5	Specify the inclusion and exclusion criteria for the review and how studies were grouped for the syntheses.	Section 2.2–2.3
Information sources	6	Specify all databases, registers, websites, organisations, reference lists and other sources searched or consulted to identify studies. Specify the date when each source was last searched or consulted.	Section 2.4 (PubMed search on 30 Sep 2025)

Section and Topic	Item #	Checklist item	Location where item is reported
Search strategy	7	Present the full search strategies for all databases, registers and websites, including any filters and limits used.	Supplementary Table S2
Selection process	8	Specify the methods used to decide whether a study met the inclusion criteria of the review, including how many reviewers screened each record and each report retrieved, whether they worked independently, and if applicable, details of automation tools used in the process.	Section 2.5
Data collection process	9	Specify the methods used to collect data from reports, including how many reviewers collected data from each report, whether they worked independently, any processes for obtaining or confirming data from study investigators, and if applicable, details of automation tools used in the process.	Section 2.6
Data items	10a	List and define all outcomes for which data were sought. Specify whether all results that were compatible with each outcome domain in each	Section 2.2 (Outcomes paragraph)

Section and Topic	Item #	Checklist item	Location where item is reported
		study were sought (e.g. for all measures, time points, analyses), and if not, the methods used to decide which results to collect.	
	10b	List and define all other variables for which data were sought (e.g. participant and intervention characteristics, funding sources). Describe any assumptions made about any missing or unclear information.	Section 2.6 (Study characteristics, covariates)
Study risk of bias assessment	11	Specify the methods used to assess risk of bias in the included studies, including details of the tool(s) used, how many reviewers assessed each study and whether they worked independently, and if applicable, details of automation tools used in the process.	Section 2.7
Effect measures	12	Specify for each outcome the effect measure(s) (e.g. risk ratio, mean difference) used in the synthesis or presentation of results.	Section 2.8 and 2.8.3
Synthesis methods	13a	Describe the processes used to decide which studies were eligible for	Section 2.2 and 2.8

Section and Topic	Item #	Checklist item	Location where item is reported
		each synthesis (e.g. tabulating the study intervention characteristics and comparing against the planned groups for each synthesis (item #5)).	
	13b	Describe any methods required to prepare the data for presentation or synthesis, such as handling of missing summary statistics, or data conversions.	Section 2.6 and 2.8
	13c	Describe any methods used to tabulate or visually display results of individual studies and syntheses.	Section 2.8 (Forest/Funnel plots, Figures 2–3, S1)
	13d	Describe any methods used to synthesize results and provide a rationale for the choice(s). If meta-analysis was performed, describe the model(s), method(s) to identify the presence and extent of statistical heterogeneity, and software package(s) used.	Section 2.8.1 and 2.8.3
	13e	Describe any methods used to explore possible causes of heterogeneity among study results (e.g. subgroup analysis, meta-	Section 2.8.2 (Subgroup and sensitivity analyses)

Section and Topic	Item #	Checklist item	Location where item is reported
		regression).	
	13f	Describe any sensitivity analyses conducted to assess robustness of the synthesized results.	Section 2.8.2 (Leave-one-out, NOS < 7 exclusion)
Reporting bias assessment	14	Describe any methods used to assess risk of bias due to missing results in a synthesis (arising from reporting biases).	Section 2.8.4 (Egger's test, trim-and-fill)
Certainty assessment	15	Describe any methods used to assess certainty (or confidence) in the body of evidence for an outcome.	Section 2.7 and 3.4 (interpretation via NOS and heterogeneity)
RESULTS			
Study selection	16a	Describe the results of the search and selection process, from the number of records identified in the search to the number of studies included in the review, ideally using a flow diagram.	Section 3.1; Figure 1 (PRISMA Flow)
	16b	Cite studies that might appear to meet the inclusion criteria, but which were excluded, and explain why they were excluded.	Section 3.1 (paragraph 2: reasons for exclusion)
Study characteristics	17	Cite each included study and present its characteristics.	Section 3.2; Table 1

Section and Topic	Item #	Checklist item	Location where item is reported
Risk of bias in studies	18	Present assessments of risk of bias for each included study.	Section 3.2 (NOS summary); Supplementary Table S3
Results of individual studies	19	For all outcomes, present, for each study: (a) summary statistics for each group (where appropriate) and (b) an effect estimate and its precision (e.g. confidence/credible interval), ideally using structured tables or plots.	Section 3.3–3.4; Figures 2–3
Results of syntheses	20a	For each synthesis, briefly summarise the characteristics and risk of bias among contributing studies.	Section 3.2; first paragraph of 3.3
	20b	Present results of all statistical syntheses conducted. If meta-analysis was done, present for each the summary estimate and its precision (e.g. confidence/credible interval) and measures of statistical heterogeneity. If comparing groups, describe the direction of the effect.	Section 3.3–3.4 (Figures 2–3)
	20c	Present results of all investigations of possible causes of heterogeneity among study results.	Section 3.5 (Meta-regression results)

Section and Topic	Item #	Checklist item	Location where item is reported
	20d	Present results of all sensitivity analyses conducted to assess the robustness of the synthesized results.	Section 3.5 (Leave-one-out, NOS < 7)
Reporting biases	21	Present assessments of risk of bias due to missing results (arising from reporting biases) for each synthesis assessed.	Section 3.5 (Egger's test, funnel plot symmetry)
Certainty of evidence	22	Present assessments of certainty (or confidence) in the body of evidence for each outcome assessed.	Section 3.6 (Summary of findings)
DISCUSSION			
Discussion	23a	Provide a general interpretation of the results in the context of other evidence.	Section 4.1–4.2
	23b	Discuss any limitations of the evidence included in the review.	Section 4.3
	23c	Discuss any limitations of the review processes used.	Section 4.3 (paragraph 2–3)
	23d	Discuss implications of the results for practice, policy, and future research.	Section 4.4–4.6
OTHER INFORMATION			
Registration and protocol	24a	Provide registration information for the review, including register name and registration	Section 2.1 (PROSPERO ID CRD420251170644)

Section and Topic	Item #	Checklist item	Location where item is reported
		number, or state that the review was not registered.	
	24b	Indicate where the review protocol can be accessed, or state that a protocol was not prepared.	Section 2.1 (PROSPERO registration details)
	24c	Describe and explain any amendments to information provided at registration or in the protocol.	Section 2.1 ("No amendments were made")
Support	25	Describe sources of financial or non-financial support for the review, and the role of the funders or sponsors in the review.	Funding section: "This research received no external funding."
Competing interests	26	Declare any competing interests of review authors.	Conflicts of Interest section
Availability of data, code and other materials	27	Report which of the following are publicly available and where they can be found: template data collection forms; data extracted from included studies; data used for all analyses; analytic code; any other materials used in the review.	Data Availability Statement: "All data are available within published articles (refs [15–29])."

Supplementary Table S2. Detailed PubMed Search Strategy.

Database	Search date	Search period covered	Search query (PubMed/MEDLINE)	Records retrieved (n)	Notes
PubMed (MEDLINE)	30 September 2025	1 January 2020 – 30 September 2025	("Long COVID" OR "post-acute sequelae of SARS-CoV-2" OR "post COVID condition" OR "PASC") AND ("prospective" OR "cohort" OR "longitudinal" OR "follow-up") AND ("prevalence" OR "risk factors" OR "outcomes") AND ("2020/01/01"[Date - Publication] : "2025/09/30"[Date - Publication])	150	No language or publication-type filters were applied. Reference lists of included studies and relevant reviews were manually screened to identify additional eligible studies. No gray literature or registry data were included.

Supplementary Table S2. Detailed PubMed search strategy used to identify prospective cohort studies on Long COVID (PASC) prevalence and risk factors. The search combined MeSH and free-text terms related to Long COVID, cohort design, and outcomes. The final search was performed on 30 September 2025, yielding 150 records, all from PubMed/MEDLINE.

Supplementary Table S3. Newcastle–Ottawa Scale (NOS) assessment of methodological quality for the 14 included prospective cohort studies on Long COVID (PASC). Most studies scored between 7 and 9 points (median = 8), indicating predominantly *low risk of bias*. Common limitations included incomplete follow-up in four studies (Naik S, Kim Y, Seeßle J, Xie Y) and reliance on self-reported outcomes in three (Kim Y, Wentz E, Seeßle J).

First Author (Year)	Country	Selection (max 4)	Comparability (max 2)	Outcome (max 3)	Total NOS Score (max 9)	Risk of Bias Interpretation
Naik S (2021)	India	4	2	2	8	Low

First Author (Year)	Country	Selection (max 4)	Comparability (max 2)	Outcome (max 3)	Total NOS Score (max 9)	Risk of Bias Interpretation
Kim Y (2023)	South Korea	3	2	2	7	Low
Frontera J (2021)	USA	4	2	2	8	Low
Del Brutto O (2021)	Ecuador	3	2	2	7	Low
Joseph G (2024)	Israel	4	2	3	9	Low
Wentz E (2024)	USA	4	2	3	9	Low
Pasculli P (2024)	Italy	3	2	2	7	Low
Kama S (2025)	France	4	2	3	9	Low
Santa Cruz A (2023)	Brazil	4	2	2	8	Low
Wu X (2021)	China	4	2	2	8	Low
Huang L (2022)	China	4	2	3	9	Low
Fischer A (2025)	Luxembourg	4	2	2	8	Low
Seeßle J (2022)	Germany	3	2	2	7	Low
Xie Y (2024)	USA	4	2	3	9	Low
